# Unusual Relapse of Chronic Lymphocytic Leukemia After Remission

**DOI:** 10.7759/cureus.2176

**Published:** 2018-02-09

**Authors:** Wajeeha Rizvi, Quoc Truong

**Affiliations:** 1 Internal Medicine, University of Kansas School of Medicine - Wichita

**Keywords:** cll-chronic lymphocytic leukemia, ibrutinib, leptomeningeal disease, cns malignancies, cancer relapse

## Abstract

Chronic lymphocytic leukemia (CLL) is the most prevalent leukemia with over 20,000 estimated cases in 2017. Leukemic involvement of the nervous system from CLL causing neurologic symptoms is reported in only about one percent of patients. Unfortunately, there is no current standard therapy for the treatment of CLL leptomeningeal disease. In this case, we discuss an unusual presentation of CLL leptomeningeal disease misdiagnosed as chronic rebound headache.

A 61-year-old female was diagnosed with Rai stage I CLL in 2002. She presented at that time with peripheral blood lymphocytosis and subsequent flow cytometry revealed a mature B cell population consistent with CLL. She was monitored clinically as there were no indications for therapy. In 2006, she developed B symptoms along with hemolytic anemia refractory to steroids and was initiated on chemotherapy with fludarabine, cyclophosphamide, and rituximab (FCR). She had a complete response after six cycles.

The patient was in her usual state of health until 2016, when she complained of chronic headaches. She took acetaminophen and ibuprofen regularly and was diagnosed with rebound headaches by neurology. These symptoms progressed and the patient developed encephalopathy requiring inpatient admission. Magnetic resonance imaging (MRI) revealed abnormal enhancement in the cerebellar peduncles and dentate nuclei symmetrically; a lumbar puncture performed revealed evidence of CLL consistent with leptomeningeal disease. Therapy was started with oral ibrutinib at 560 mg daily for better central nervous system (CNS) penetration. After three months of therapy, she had complete resolution of symptoms and MRI abnormalities.

Leptomeningeal disease is a rare complication of CLL that clinicians should be aware of and ibrutinib can be an effective, tolerable therapy for this debilitating disease.

## Introduction

In the western world, chronic lymphocytic leukemia (CLL) is the most prevalent leukemia with estimated cases in 2017 exceeding 20,000 patients [1-2]. CLL is a known heterogeneous disease, which presents with a wide spectrum of clinical symptoms at diagnoses and progression. Leukemic involvement of the nervous system from CLL causing neurologic symptoms occurs infrequently and has been reported in only one percent of patients with CLL [3]. Reske-Nielsen et al. first discovered it in 1974. Unfortunately, there is no current standard protocol for the treatment of CLL leptomeningeal disease. In this case, we discuss an unusual presentation of CLL leptomeningeal disease misdiagnosed as chronic rebound headache responding well to oral ibrutinib therapy.

## Case presentation

This is a 61-year-old female who was diagnosed with Rai stage I CLL in 2002. She presented with peripheral blood lymphocytosis and subsequent flow cytometry analysis revealed a phenotype of a mature B cell population showing kappa light chain restriction, expressing CD5 and CD23 consistent with CLL. The patient was monitored clinically at that time, as she did not meet criteria for therapy. The patient’s white blood cells (WBC) progressed slowly from 17,800 to approximately 50,000/cmm by the end of 2005; in 2006, she developed B symptoms consisting of night sweats, fatigue with splenomegaly, and diffuses lymphadenopathy. She subsequently developed haemolytic anemia refractory to steroids and was initiated on chemotherapy with fludarabine, cyclophosphamide, and rituximab (FCR) chemotherapy. The patient tolerated chemotherapy well and had a complete response after six cycles of FCR.

**Figure 1:**
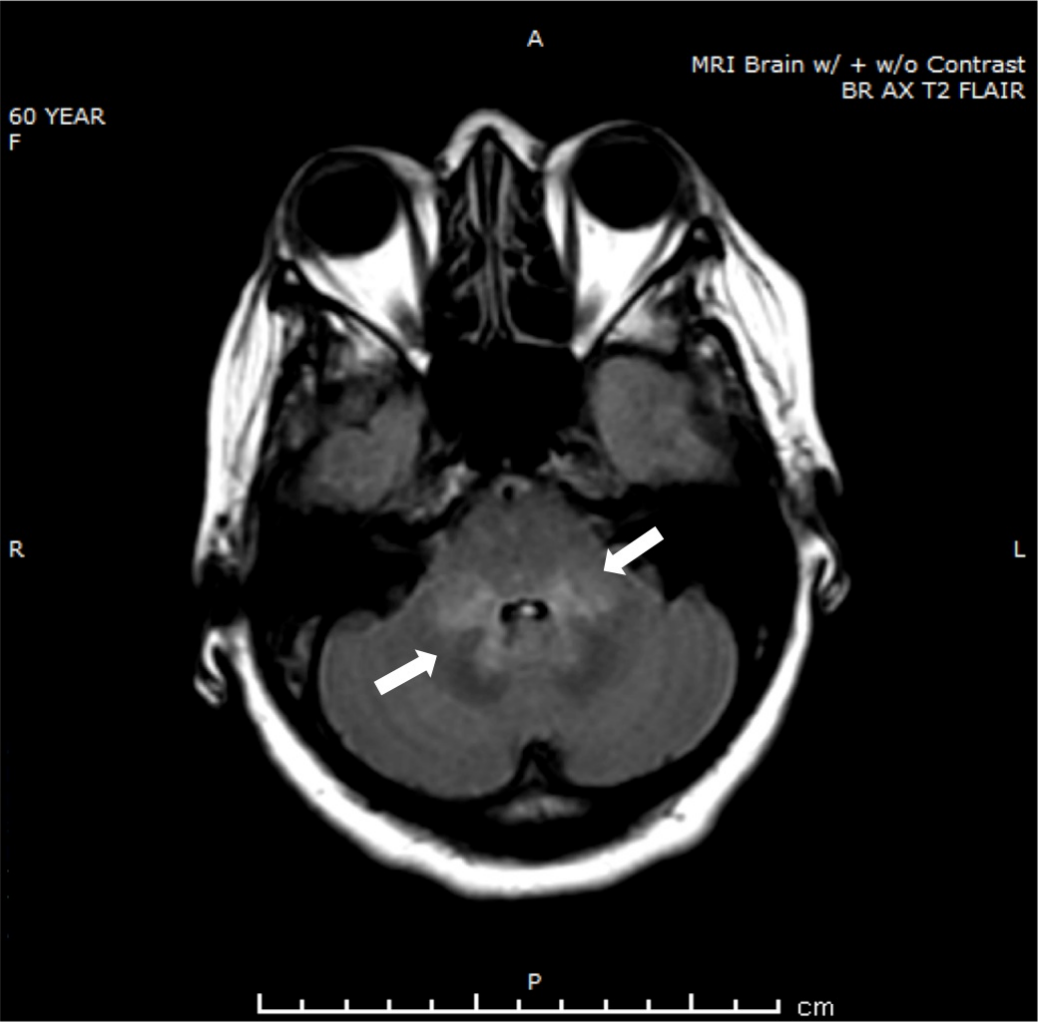
Magnetic resonance imaging at the time of presentation with headaches

The patient was in her usual state of health until 2016, when she complained of chronic headaches. She started taking acetaminophen and ibuprofen on a daily basis and subsequently was evaluated by Neurology and diagnosed with rebound headaches. These symptoms progressed until she was admitted to the hospital for mental status changes and subsequently was evaluated by Oncology. A magnetic resonance imaging (MRI) brain scan revealed signal changes in the cerebellar peduncles and dentate nuclei symmetrically as shown in Figure 1.

**Figure 2:**
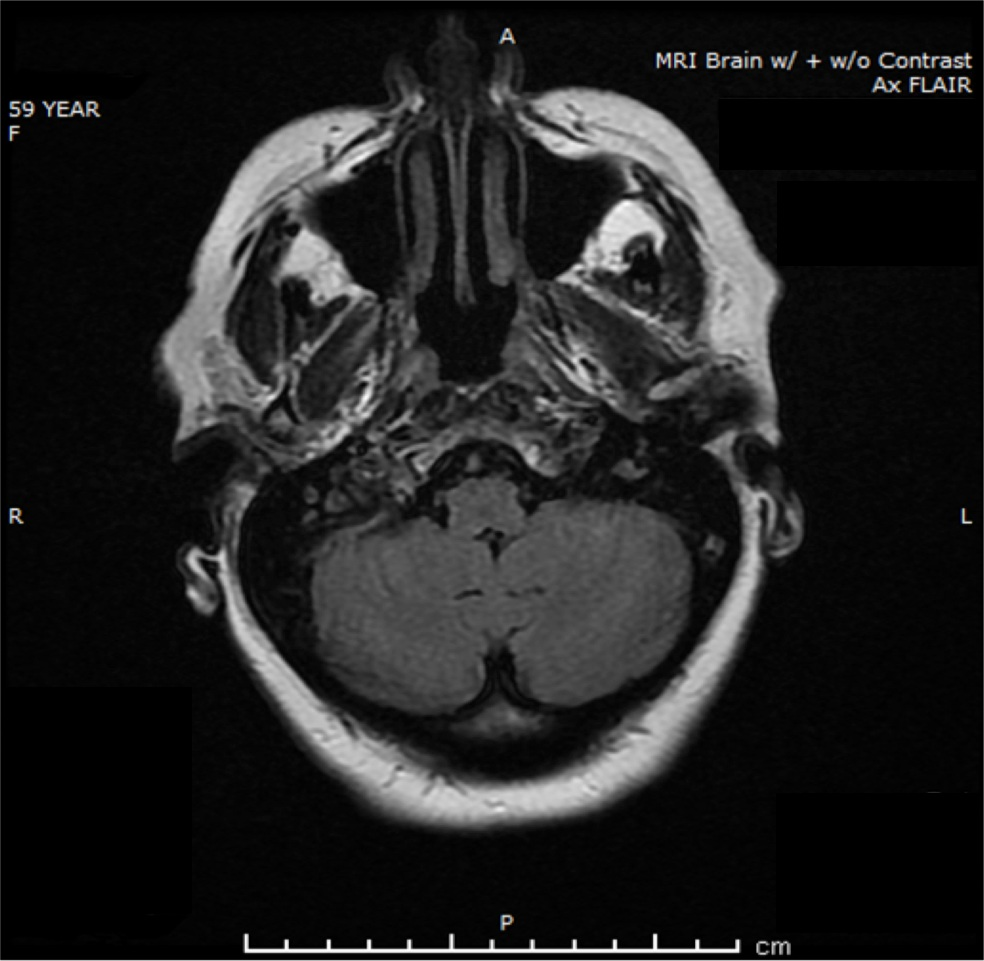
Magnetic resonance imaging one year ago, at the time of remission

This finding was not present on the MRI brain done in 2015, as shown in Figure 2. At that time, there was no enhancing lesion or hemorrhage noted.

Lumbar puncture was completed and revealed evidence of CLL involving central nervous system (CNS) consistent with leptomeningeal disease. Richter transformation was ruled out. She did not have any other evidence of systemic disease; laboratory including WBC was otherwise normal. Therapy was discussed with the patient to consider intrathecal chemotherapy versus oral ibrutinib, which has known activity in the CNS. The patient was initiated on ibrutinib at 420 mg po daily for two weeks and subsequently increased up to 560 mg po daily. After six months of therapy, the headaches resolved. She is doing well on ibrutinib with no obvious side effects.

## Discussion

CNS involvement in CLL is extremely rare. In our literature review, we came across several studies that discussed the incidence of leptomeningeal disease in CLL. In 2016, a study reported the presence of neurological symptoms secondary to leptomeningeal disease in 18 out of 4174 CLL patients, who were followed from 1999-2014 [4].

At present, the optimal therapeutic approach is still unknown for CNS disease in relapsed CLL. Current therapies include intrathecal chemotherapy with cytotoxic agents like methotrexate and cytarabine, with or without steroids.

The treatment of relapsed CLL has been revolutionized with the advent of oral inhibitors of B-cell receptor (BCR) signal transduction [5-6]. Ibrutinib is one of the irreversible Bruton's tyrosine kinase (BTK) inhibitor, which leads to deactivation of microenvironment survival signaling and pro-survival pathways [7]. It works as a small-molecule inhibitor of BTK by forming a covalent bond with the cysteine residue in the BTK active site, causing sustained inhibition of BTK enzymatic activity. Since BTK is a signaling molecule of the BCR and cytokine receptor pathways, inhibition of this pathway disrupts B-cell trafficking, chemotaxis, and adhesion.

Ibrutinib is an orally absorbed drug that can cross the blood-brain barrier and has shown to achieve clinically significant concentration in the cerebral spinal fluid (CSF) [8]. Ibrutinib exposure increases with doses up to 840 mg.

The role of ibrutinib has been widely recognized in the treatment of mantle cell lymphoma, Waldenström macroglobulinemia, small lymphocytic lymphoma, and CLL.

Ibrutinib provides progression-free-survival with good efficacy and response. Few adverse events continue to be a barrier in its widespread use including gastrointestinal side effects, muscle cramps, and less common side effects such as atrial fibrillation, bleeding, and infectious complications.

## Conclusions

In conclusion, leptomeningeal disease is a rare complication of CLL that clinicians should be aware of. This case demonstrates that it can occur in the absence of other systemic symptoms and normal WBC count. Ibrutinib can be an effective, tolerable therapy in patients with CLL leptomeningeal disease.
